# Mapping of MPH programs in terms of geographic distribution across various universities and institutes of India—A desk research

**DOI:** 10.3389/fpubh.2024.1443844

**Published:** 2024-08-07

**Authors:** Pooja S. Dhagavkar, Mubashir Angolkar, Jyoti Nagmoti, Sanjay Zodpey

**Affiliations:** ^1^Department of Public Health, Jawaharlal Nehru Medical College, Belagavi, Karnataka, India; ^2^Department of Microbiology, Jawaharlal Nehru Medical College, Belagavi, Karnataka, India; ^3^Public Health Foundation of India, Gurugram, Haryana, India

**Keywords:** MPH, public health, education, program, map

## Abstract

**Background:**

Landscaping studies related to public health education in India do not exclusively focus on the most common Masters of Public Health (MPH) program. The field of public health faces challenges due to the absence of a professional council, resulting in fragmented documentation of these programs. This study was undertaken to map all MPH programs offered across various institutes in India in terms of their geographic distribution, accreditation status, and administration patterns.

**Methodology:**

An exhaustive internet search using various keywords was conducted to identify all MPH programs offered in India. Websites were explored for their details. A data extraction tool was developed for recording demographic and other data. Information was extracted from these websites as per the tool and collated in a matrix. Geographic coordinates obtained from Google Maps, and QGIS software facilitated map generation.

**Results:**

The search identified 116 general and 13 MPH programs with specializations offered by different universities and institutes across India. India is divided into six zones, and the distribution of MPH programs in these zones is as follows, central zone has 20 programs; the east zone has 11; the north zone has 35; the north-east zone has 07; the south zone has 26; and the west zone has 17 MPH programs. While 107 are university grants commission (UGC) approved universities and institutes, only 46 MPH programs are conducted by both UGC approved and National Assessment and Accreditation Council (NAAC) accredited universities and institutes. Five universities are categorized as central universities; 22 are deemed universities; 51 are private universities; and 29 are state universities. Nine are considered institutions of national importance by the UGC, and four institutions are recognized as institutions of eminence. All general MPH programs span 2 years and are administered under various faculties, with only 27 programs being conducted within dedicated schools or centers of public health.

**Conclusion:**

The MPH programs in India show considerable diversity in their geographic distribution, accreditation status, and administration pattern.

## Background

Public health was defined as “the science and art of preventing disease, prolonging life and promoting, protecting and improving health through the organized efforts of society” by Sir Donald Acheson in 1988 (1). Public health professional is “a person educated in public health or a related discipline who is employed to improve health through a population focus” (2). They constitute a specialized workforce with specialized training and expertise in public health, dedicating their efforts exclusively to this field. In 1946, the Bhore Committee recognized inadequacies in teaching preventive medicine and public health to medical undergraduate students. Their report recommended the inclusion and enhancement of the public health workforce in India (3). Subsequently, in 1961, the Mudaliar Committee advocated for the establishment of schools of public health in each state to bolster the education and training of this workforce. These initiatives although restricted only for medical doctors laid the foundation for the development of public health education and training programs in India (4). In 2012, a significant step toward expanding public health education beyond medical personnel was taken by the High-Level Expert Group for Universal Health Coverage. They suggested the introduction of post-graduate courses in public health for healthcare as well as non-healthcare professionals and the establishment of new public health management institutions, thus transforming public health into a transdisciplinary field (5, 6). Further, in 2017, the National Health Policy (NHP) envisioned the creation of a multidisciplinary Public Health Management Cadre (PHMC) to strengthen India's public health delivery system (7). The policy outlined strategies for attracting young, talented multidisciplinary experts to pursue careers in public health, thereby addressing the growing demand for skilled professionals in this field (8).

In India, undergraduate public health education started a decade back in the year 2013, with the first B.Sc. in public health degree course offered in Jodhpur (9). Till then public health education primarily revolved around the Master of Public Health (MPH) program, which has gained prominence over the years (10). Initially restricted only to medical graduates and offered primarily in medical colleges, MPH programs have expanded to include graduates from diverse backgrounds, reflecting the interdisciplinary nature of modern public health education (11). The first MPH program in India was introduced at Mahatma Gandhi University in 1995. In the academic year 2016–2017, 44 institutions offered MPH programs (12). In 2020, the COVID-19 pandemic underscored the critical importance of public health, highlighting the need for a well-prepared, competent public health workforce and capacity building (13). Innovative teaching-learning strategies in public health education are required to meet the evolving challenges in both the governmental and private healthcare sectors (14). Since last 5 years, the number of MPH programs offered in India has almost doubled, with more than 110 institutions today. As the demand for public health professionals increases every year, MPH programs are being introduced, especially by private institutions.

Various studies have been conducted to landscape public health education in India (6, 15–17). These studies look into the spread of all programs in public health including bachelors and masters of sciences (B.Sc. and M.Sc.), diploma as well as doctorate in public health. Despite the growing popularity of the MPH program and the increasing number of institutions offering them, there is a dearth of comprehensive studies focusing exclusively on the masters program (6, 16, 17). Given the absence of a professional council for public health education, there is no system for registering or recording MPH programs. Listing and cataloging the MPH programs in India becomes difficult. There is a lack of information on the total number, location, and other details of these programs. The study addresses this dearth of information by examining the geographic distribution, accreditation status, and administration patterns of MPH programs across various institutes in India.

## Materials and methods

Desk research was conducted from July 2023 to January 2024. Internet was searched using the Google web browser. Keywords such as “MPH,” “Masters of Public Health,” “Schools of Public Health,” “Public Health Institutions,” “Public Health Programs,” “Institutions Offering MPH,” “Public Health Specialization,” “Post Graduate MPH Courses,” and “India” were utilized to identify universities and institutions. Websites of the University Grants Commission (UGC) and other education-related platforms were looked up. Previous publications (12, 16–19) pertaining to public health education in India were referred to supplement the data gathering process. Any duplicate entries were eliminated. The search included all universities and institutions currently offering MPH programs in India. Institutions that offered only MPH specializations and MPH honors degrees were identified separately. This meticulous approach led to the compilation of a comprehensive state-wise catalog of universities and institutions offering MPH programs in India.

To facilitate data collection, a structured data extraction tool was developed that captured information on demographic variables like state wise location and exact address of the universities and institutions. It also captured information pertinent to accreditation status, and administration patterns of MPH programs such as university affiliation, National Assessment and Accreditation Council (NAAC) accreditation status, UGC approval and classification, duration of the MPH program, discipline under which the program is conducted, presence of a dedicated school or department of public health, as well as any national and international collaborations. The websites of all the cataloged universities and institutions were thoroughly explored for details pertaining to their MPH programs. Data from these websites was extracted as per the tool. In cases where websites provided incomplete information, respective program coordinators were contacted for clarification. Publicly available MPH program brochures and prospectuses were reviewed to extract information. The extracted data were organized and tabulated in an Excel matrix for further analysis and interpretation.

Geographical coordinates (latitudes and longitudes) of the identified universities and institutes were obtained from Google Maps based on the address information provided on their respective websites. These coordinates were then utilized in the QGIS version 3.34.3 software, a free and open-source geographic information system, to generate maps depicting the distribution of MPH programs across different regions and to analyze various related variables.

## Results

**Figure 1:**
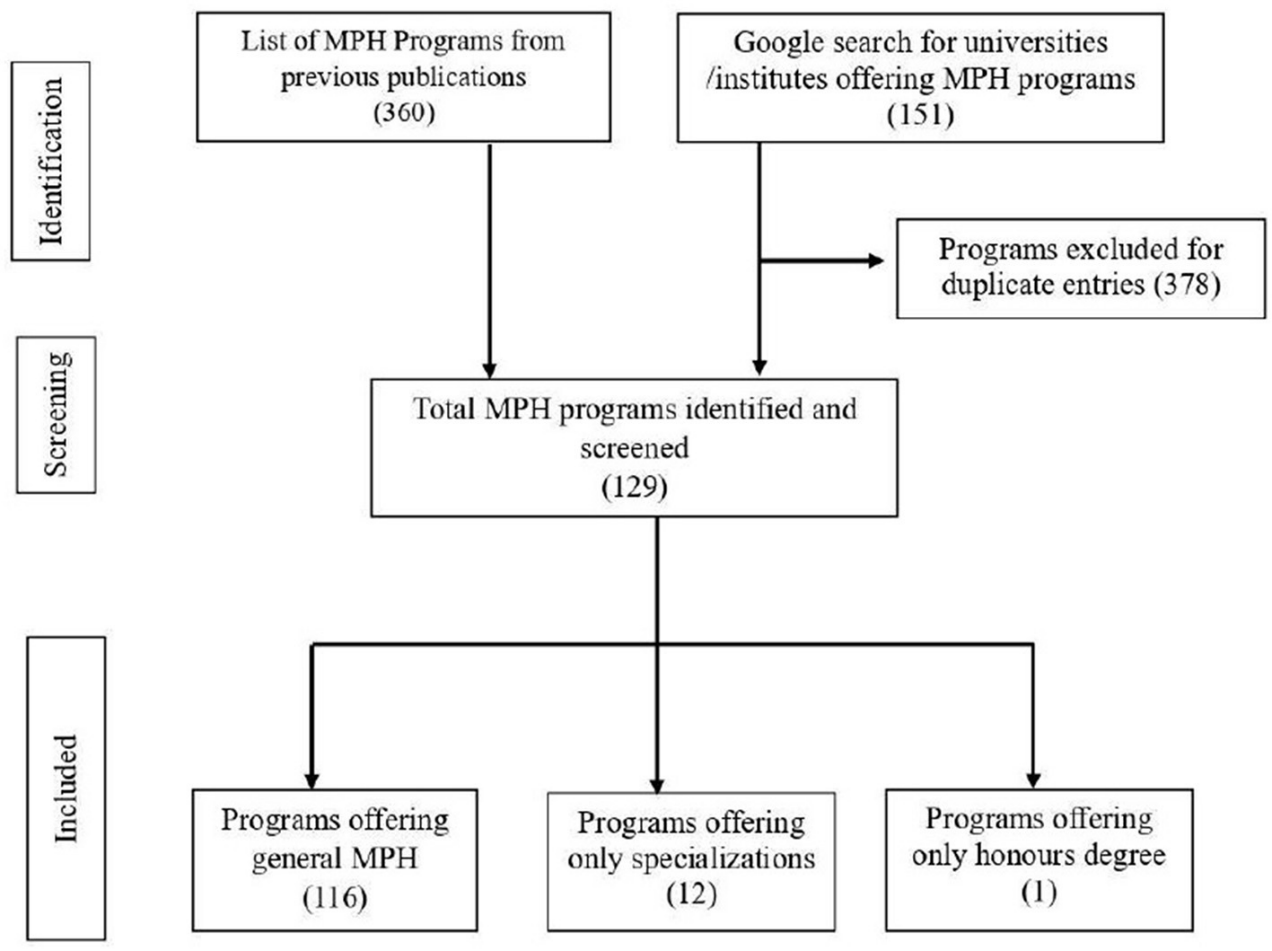
Selection of MPH programs in India.

A total of 511 MPH programs were identified through the desk review, of which 382 were duplicate entries. After filtering these duplicates, the final count of MPH programs in India stands at 129. Among the 129 programs conducted by the respective universities and institutes, 13 institutes offer only specializations in MPH. The selection process is shown in Figure 1.

All India Institute of Hygiene and Public Health Kolkata offers MD (MPH Epidemiology) for 3 years; ICMR-National Institute of Epidemiology Chennai offers both MPH (Epidemiology) and MPH (Health Systems) both for 2 years; Tata Institute of Social Sciences Mumbai offers MPH (Health Policy, Economics, and Finance), MPH (Health Administration), and MPH (Social Epidemiology) all for 2 years; 9 Government Medical Colleges in Maharashtra offer MPH (Nutrition) for 2 years; and the Institute of Public Health and Center for Disease Control affiliated to Rajiv Gandhi University of Health Sciences Karnataka offers an honors degree in MPH for 3 years duration. Therefore, 116 general MPH programs and 13 specializations in MPH are offered in India as of January 2024. Only general MPH programs were included in the final analysis. All general MPH programs have a duration of 2 years.

### Geographical distribution of general MPH programs

According to the States Reorganization Act of 1956, India established six zonal councils, namely Central, East, North, North East, South, and West. The *central zone* includes the states of Chhattisgarh, Madhya Pradesh, Uttarakhand, and Uttar Pradesh. The *east zone* includes Bihar, Jharkhand, Odisha, and West Bengal states. The *north zone* includes the states of Chandigarh, Delhi, Haryana, Himachal Pradesh, Jammu and Kashmir, Punjab, Rajasthan, and the union territory of Ladakh. The *north-east* zone includes the states of Assam, Arunachal Pradesh, Manipur, Meghalaya, Mizoram, Nagaland, Tripura, and Sikkim. The *south zone* includes the states of Andhra Pradesh, Karnataka, Kerala, Puducherry, Tamil Nadu, and Telangana, and the *west zone* includes Gujarat, Goa, and Maharashtra states and the union territories of Dadra and Nagar Haveli and Daman and Diu.

**Figure 2:**
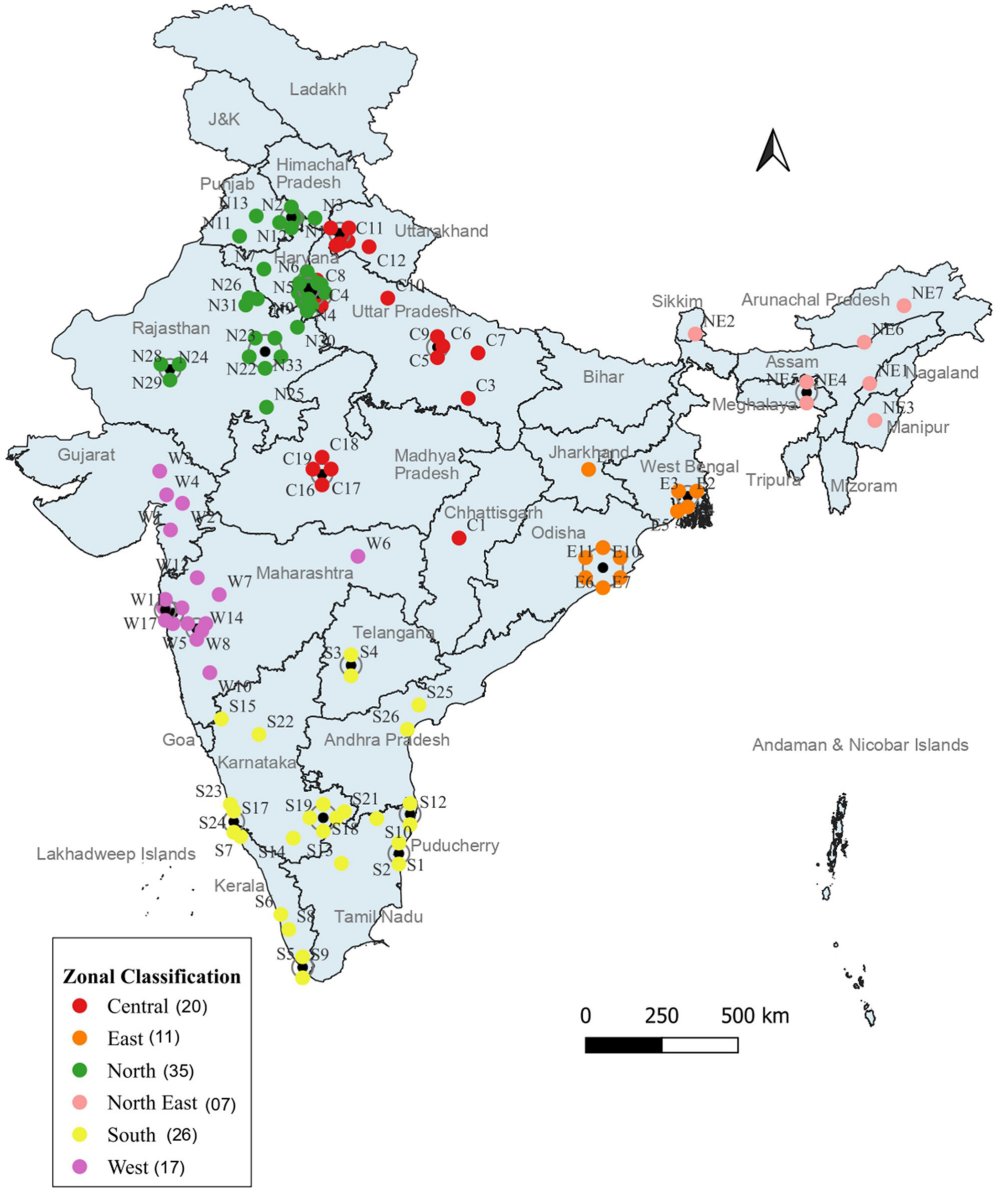
Zonal mapping of universities/institutions offering general MPH programs.

The universities and institutes offering MPH programs were therefore mapped according to these six zones. Each university or institute was assigned a unique identification code. This code consisted of a zonal code, the state code, the regional code, and the university or institute number (Supplementary Table 1). The distribution of general MPH programs across these zones is shown in Figure 2.

A significant concentration of general MPH programs is observed in the southern states of Maharashtra (13 programs) and Karnataka (11 programs), as well as in the northern state of Rajasthan (13 programs). Conversely, several states, including Assam, Bihar, Goa, Mizoram, Tripura, and the Union Territories of Andaman and Nicobar Islands, Dadar and Nagar Haveli, Jammu and Kashmir, Ladakh, Lakshadweep, and Daman and Diu, did not have any MPH programs available.

### Distribution of MPH programs according to their discipline

**Figure 3:**
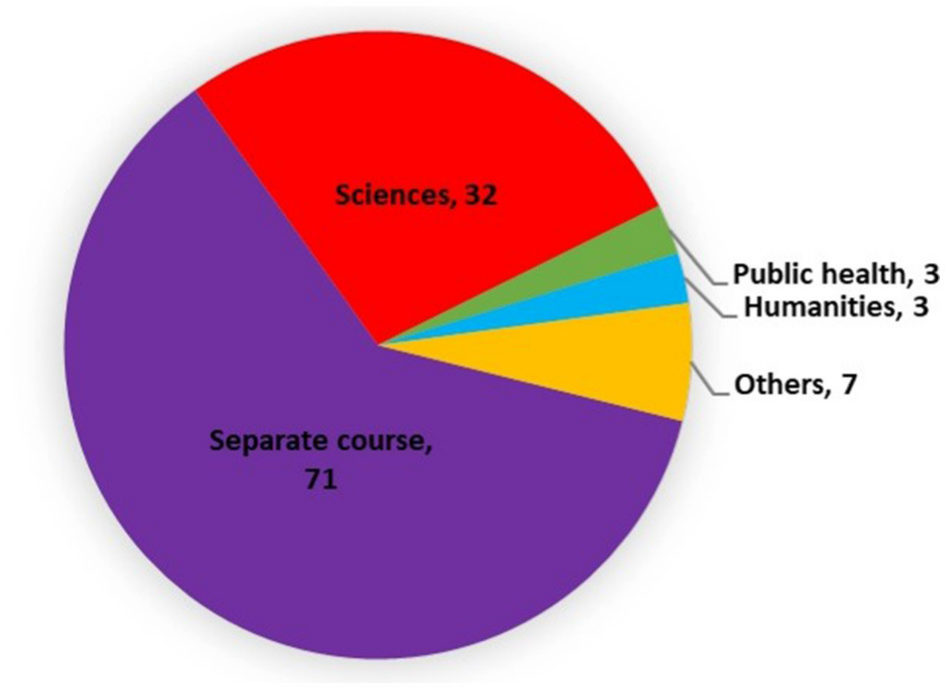
Distribution of MPH programs according to their discipline.

In India general MPH programs run under various disciplines, distribution of which is highlighted in Figure 3. Majority of these programs which are not categorized under any specific discipline, are administered as standalone courses by the respective universities or institutes. Within the science discipline, 16 programs are classified under health and allied sciences, 6 under medical and health sciences, five under paramedical sciences, and 1 each under applied life and social sciences. Additionally, three programs are conducted under the disciplines of public health and humanities, respectively.

One program is running under the disciplines of commerce and management, different areas of health, medical and mental abilities, healthcare management, hospital administration, public health, nursing, and business. Moreover, a unique program is identified as the Rural Health Postgraduate Program. These are clubbed under the others category in Figure 3.

### NAAC and UGC accreditation and UGC classification

**Figure 4:**
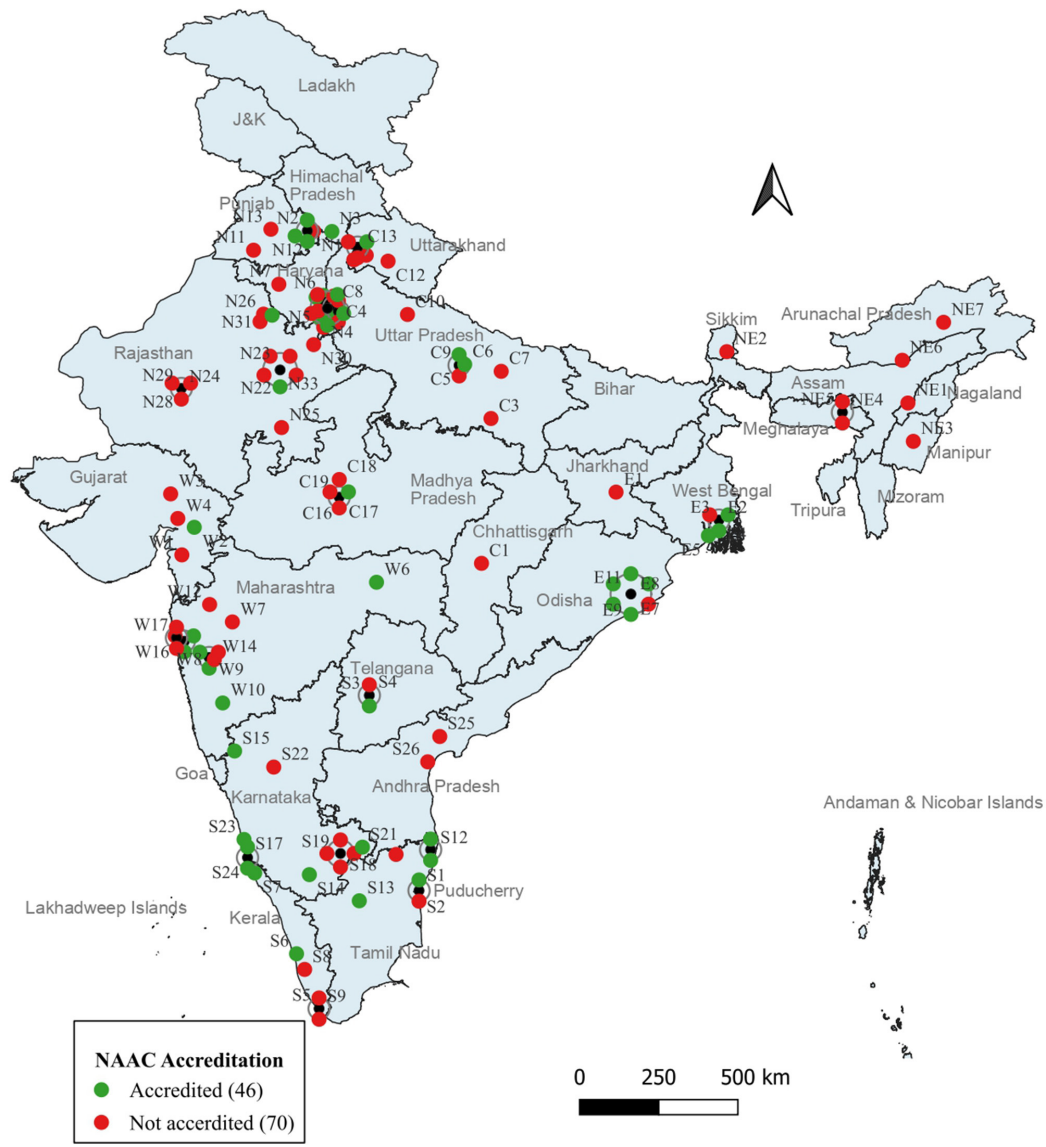
NAAC accreditation of universities/institutions offering MPH programs.

Most of the NAAC accredited universities and institutes offering general MPH programs in India lie in the north and south zones (13 each). None of the universities and institutes in the north eastern zone are NAAC accredited. The distribution is depicted in Figure 4.

Out of the 116 general MPH programs, five universities and institutions two from the north zone in the state of Delhi and one each from the south, central and west zone do not hold UGC approval.

**Figure 5:**
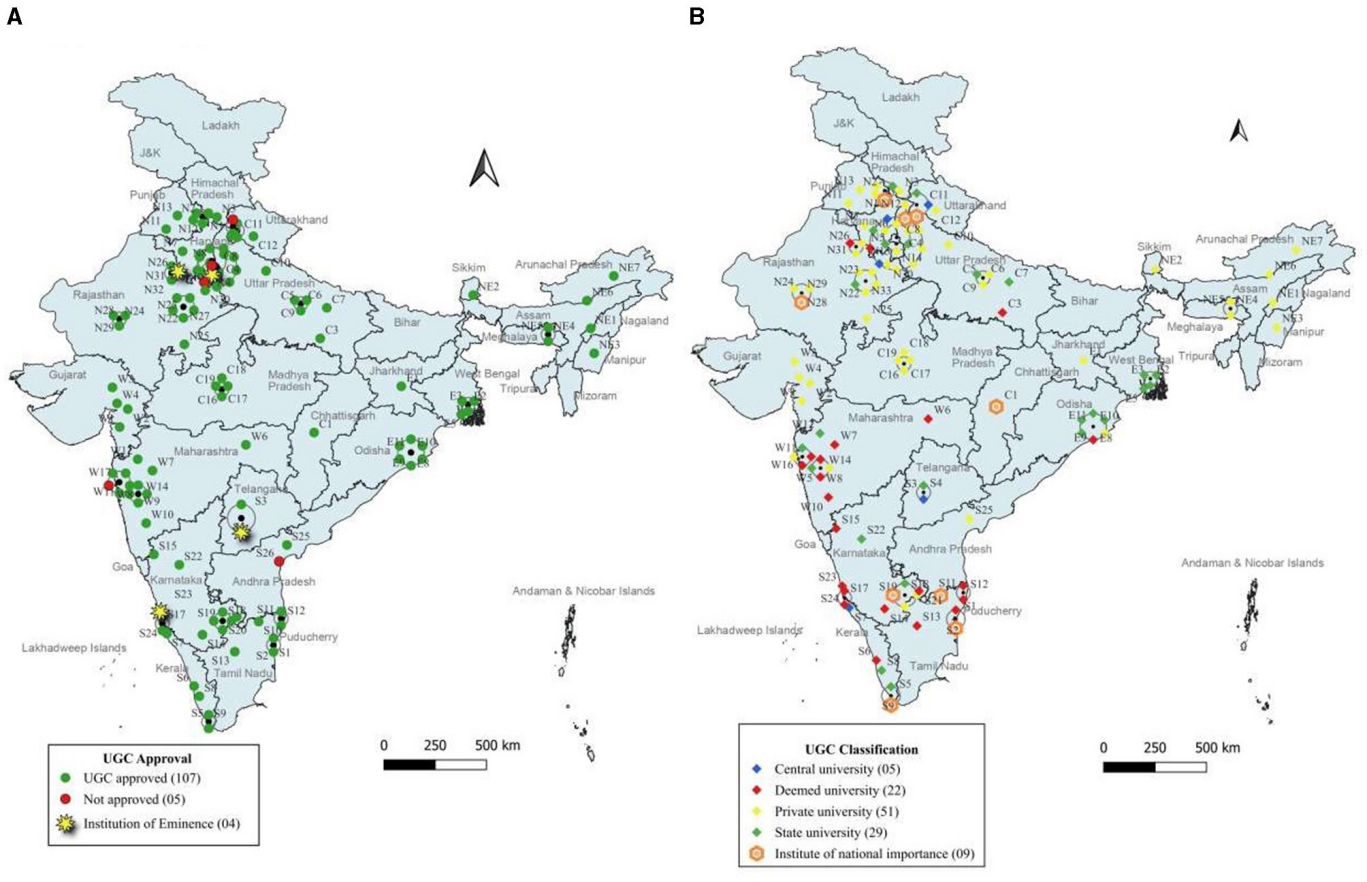
UGC approval and classification of universities/institutions offering MPH programs. **(A)** UGC approval. **(B)** UGC classification.

According to UGC, central universities are established by the Act of Parliament and are funded by the central government. Institutions of National Importance are also established by the act of parliament. State Universities are established by the Act of the State legislature concerned and are funded by the state government. Private universities are established by the Act of the State legislature concerned and are self-financing institutions. Deemed universities are declared by the notification of the government of India, on the advice of UGC, under Section 3 of the UGC Act, 1956. General MPH programs in India according to this classification is shown in Figure 5. Distribution of these programs according to NAAC accreditation and UGC classification is mentioned in Supplementary Table 1.

### Presence of dedicated schools, centers, or departments for conducting MPH programs

Among the total MPH programs, 39 are not under any dedicated department, indicating a general administrative structure. Conversely, 29 MPH programs are conducted under separate schools within the universities, with two of these schools hosting a dedicated department of public health.

Among the 27 programs conducted within dedicated schools or centers of public health, two programs are conducted under the department of epidemiology, and one program is under the department of health policy.

**Figure 6:**
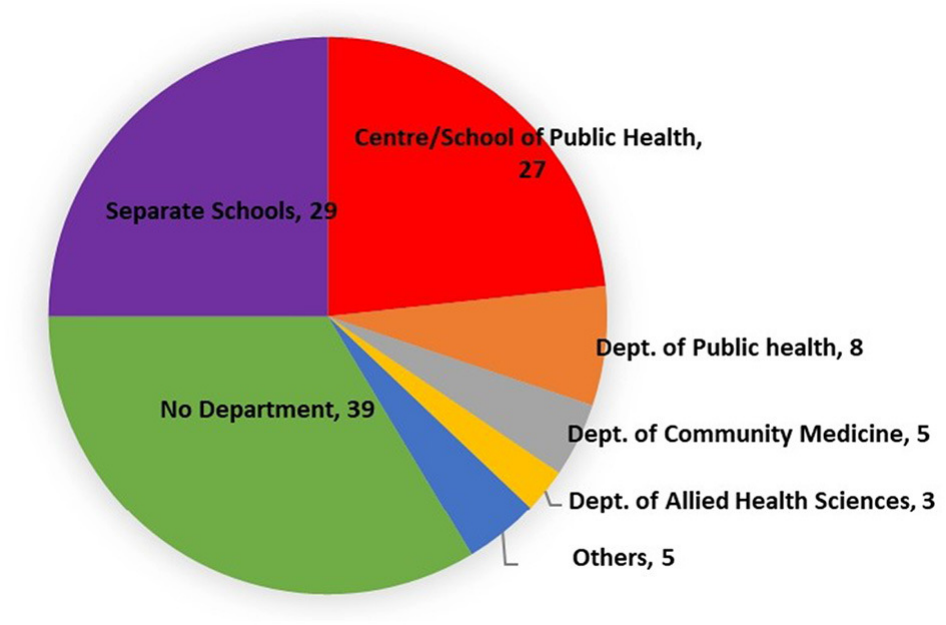
Distribution of MPH programs according to schools/centers/departments conducting them.

One MPH program each is under the departments of paramedical science, environmental health engineering, social work, management, and zoology, respectively which are clubbed in the others category. These diverse departmental affiliations showcase the multidisciplinary nature of public health education. This distribution is shown in the Figure 6.

It is noteworthy that the SD Gupta School of Public Health at IIHMR University offers an MPH program in collaboration with the Johns Hopkins Bloomberg School of Public Health, USA, highlighting international cooperation in public health education. Similarly, the University of Hyderabad's MPH program, housed within the School of Medical Sciences, is conducted in partnership with the Indian Institute of Public Health (IIPH) in Hyderabad, showcasing collaborative efforts within the national context.

## Discussion

Education in public health in India has gained significant traction. Particularly following the COVID-19 pandemic, the Master of Public Health (MPH) program has emerged as a most widely available and sought-after public health education program. However, studies indicate a notable variance in the operational frameworks of these MPH programs across India (13, 17). While this diversity fosters adaptability and innovation within the program, there is a pressing need to streamline the MPH programs throughout India in order to align it with the requirements of the National Education Policy 2020 which includes transfer of credits across the programs and multiple entry and exit through and from the programs (20). Although the model course/curriculum for MPH developed by the Ministry of Health & Welfare, Govt. of India (21) may be an initiating step toward this process, comprehending the distribution of MPH programs is imperative, forming the basis for a more profound analysis of the existing differences within the Indian MPH landscape. This study was therefore undertaken to map all MPH programs offered across various institutes in India.

The clustering of MPH programs within specific geographical zones is evidenced by this study. This raises questions regarding the absence of such programs in certain other states. According to the NITI Aayog health index 2021, Kerala, Tamil Nadu, and Telangana states ranked among the top performers, while Bihar and Uttar Pradesh were among the last two (22). In this study, the clustering of MPH programs is seen, especially in the northern and southern zones, but scarce in the others. This correlation warrants further exploration to discern underlying factors like health indices, socioeconomic development, and others influencing the distribution of public health education initiatives across different states.

All general MPH programs in India run for a duration of 2 years. Whether this timeframe adequately equips students with the requisite competencies for public health practice is a lesser- researched aspect. For this, it is imperative for public health education to be organized, the academic and work competencies to be laid down, and criteria to be established to justify their accomplishment within a structured time frame. MPH programs in India are conducted under a wide range of disciplines or as separate courses. It is understandable that, due to the diverse disciplinary backgrounds of teachers instructing in this program, the rationale for consolidating them under a single discipline may not be justified. However, it is required to streamline the disciplines based on a consensus from stakeholders in the field of public health.

As seen in this study, almost all institutions, except five offering MPH programs in India, are UGC- approved. The universities established by central (an act of the parliament) or state Government are valid universities as per the UGC. The NAAC is an autonomous institution established by the UGC. In India, all higher education institutions are required to undergo UGC assessment and accreditation by the NAAC. Institutions of public health education in India are eligible to apply for accreditation and assessment from the NAAC, but majority of them are not NAAC accredited. Accreditation bodies play a crucial role in enabling universities and institutions to identify their strengths and opportunities while addressing their weaknesses and challenges. Additionally, accreditation provides society with trustworthy information regarding the quality of education offered by these institutions (23). However, there is a notable lack of literature on the quality of Master of Public Health (MPH) programs and the impact of accreditation on this quality. The question of why some universities in a state pursue accreditation while others do not remain unanswered. Consequently, further research is needed to explore this issue comprehensively.

The South-East Asia Public Health Education Institutes Network (SEAPHEIN) has devised strategies to develop institutional accreditation mechanisms for public health education in the region (23). The Indian chapter of SEAPHEIN, the India Public Health Education Institution Network (IndiaPHEIN), was established in July 2010 with a mission to collaborate with Indian member institutes to improve and sustain the quality and relevance of public health education (24). Despite these efforts, the accreditation framework for Master of Public Health (MPH) programs in India remains to be developed. Although accreditation information is typically displayed on the websites of universities and institutions, it may go unnoticed if not adequately emphasized. Furthermore, there is no centralized documentation of the accreditation status of institutions offering MPH programs. This lack of centralization may contribute to the majority of these institutions remaining unaccredited. The absence of a professional council further compounds this challenge. The Public Health Academic Ranking (PHAR) represents a novel model designed to establish a global ranking for schools of public health (25). This ranking system is based exclusively on bibliometric indicators that assess the research domain of public health, without evaluating the areas of education and teaching. Currently, the PHAR is undergoing refinement and improvements. Incorporating domains such as public health education into this ranking system could enhance its utility, potentially facilitating the accreditation process for public health schools and, consequently, Master of Public Health (MPH) programs in India.

In India, the departments of “Community Medicine” have historically been associated with public health education (26). Programs offered by these departments are open only for medical graduates and not for non-medical graduates like those from the fields of social sciences, life sciences or management. As seen in this study on the present date MPH programs are functional under various departments in India. Each department concentrates on teaching the program based on their organizational objectives, strengths of their faculty, etc. Thus, some programs may focus on social sciences, some on epidemiology, some on mental health and likewise. The curriculum taught changes accordingly. Understanding this distribution will help stakeholders choose programs according to their area of interest. This being said, public health is a multidisciplinary and transdisciplinary profession with a variable degree of inclusivity. Hence, the nuances required in this profession can be understood only by core public health professionals. It is important for this reason to run MPH programs under an uniform banner of a public health school or a public health department to strengthen public health as an independent discipline.

The biggest limitation of this study was direct engagement with program coordinators. Similarly, analysis of core competencies addressed by the MPH programs would give a better understanding of their differences in terms of administration of the curricular framework. This highlights a potential avenue for future research to validate findings and delve deeper into the design, implementation, standardization, and revision processes for these programs. Further studies are required to assess the quality of MPH programs offered in India.

## Conclusion

Previous studies have shown that the number of universities and institutes offering MPH programs in India has surged since 1995, reaching 116 general and 13 specializations in MPH programs by January 2024. However, these programs exhibit considerable diversity in terms of their geographic distribution, accreditation status, and administrative structures. Predominantly, these MPH programs are concentrated in the northern and southern regions of India. While most of the universities and institutes offering these programs in India hold UGC approval, NAAC accreditation remains uncommon. According to the UGC, these universities and institutes are classified as central, state, private, and deemed. It would be interesting to see whether this classification has any impact on the implementation of these programs in future studies. Indian MPH programs are offered either as stand-alone programs by different universities or under a wide range of disciplines. By mapping MPH programs, stakeholders can gain a clearer understanding of the current landscape and identify disparities thus helping them with informed decision making and work toward creating a more equitable public health education system in India.

## Data availability statement

The original contributions presented in the study are included in the article/Supplementary material, further inquiries can be directed to the corresponding author.

## Author contributions

PD: Conceptualization, Data curation, Formal analysis, Investigation, Methodology, Software, Visualization, Writing – original draft, Writing – review & editing. MA: Conceptualization, Methodology, Supervision, Writing – review & editing. JN: Project administration, Supervision, Writing – review & editing. SZ: Conceptualization, Methodology, Project administration, Supervision, Writing – review & editing.
