# Supplementary material for: Mapping of MPH programs in terms of geographic distribution across various universities and institutes of India—A desk research
Source: Front Public Health. 2024 Aug 7;12:1443844. doi: 10.3389/fpubh.2024.1443844 (PMC11335718; doi:10.3389/fpubh.2024.1443844)
Supplement: Supplementary file 1 [file Data_Sheet_1.PDF]

Supplementary Material

Table No. 1. Unique Identification Codes for Universities and Institutes Offering MPH Programs In India

Zones	Unique Identification Code	Name of Universities/Institutes Offering MPH Programs in India	City/ District	State/ Union Territory	NAAC accreditation	UGC Classification
NORTH	NCHC1	Postgraduate Institute of Medical Education and Research (PGIMER)	-	Chandigarh	Not Accredited	UGC approved Institute of National Importance
	NCHC2	University Institute for Emerging Areas in Science & Technology (UIEAST)	-	Chandigarh	Accredited	State university
	NHPS3	Akal College of Health and Allied Sciences	Sirmaur district	Himachal Pradesh	Accredited	Private university
	NHRG4	GD Goenka University	Gurugram	Haryana	Not Accredited	Private university
	NHRG5	Amity Medical School	Gurugram	Haryana	Accredited	Private university
	NHRS6	Jindal School of Public Health and Human Development	Sonipat	Haryana	Accredited	Private university
	NHRH7	OM Sterling Global University	Hisar	Haryana	Not Accredited	Private university
	NHRB8	Gurugram University	Gurugram	Haryana	Accredited	State university
	NHRG9	Shree Guru Gobind Singh Tricentenary University	Gurugram	Haryana	Not Accredited	Private university
	NPBS10	Rayat Bahra University	Sahibzada Ajit Singh Nagar (Mohali)	Punjab	Accredited	Private university
	NPBB11	Adesh Institute of Medical Sciences and Research	Bhatinda	Punjab	Not Accredited	Private university
	NPBF12	Desh Bhagat University	Fatehgarh Sahib	Punjab	Accredited	Private university
	NPBL13	CT University	Ludhiana	Punjab	Not Accredited	Private university
	NDLG14	Indian Institute of Public Health Delhi	-	Delhi	Not Accredited	UGC approved

						Institute of National Importance
	NDLSWD15	Jawaharlal Nehru University	-	Delhi	Accredited	Central university
	NDLSWD16	School of Allied Health Sciences and Management	-	Delhi	Not Accredited	State university
	NDLSD17	Hamdard Institute of Medical Science and Research	-	Delhi	Accredited	Deemed university
	NDLSD18	ICRI- Gurugram campus	-	Delhi	Not Accredited	Private university
	NDLND19	National Institute of Health and Family Welfare	-	Delhi	Accredited	Central university
	NDLWD20	Ambedkar University	-	Delhi	Accredited	State university
	NRJJA21	SD Gupta School of Public Health	Jaipur	Rajasthan	Accredited	Private university
	NRJJA22	School of Humanities, Social Science and Fine Arts	Jaipur	Rajasthan	Not Accredited	Private university
	NRJJA23	Mahatma Jyoti Rao Phoole University (MJRP)	Jaipur	Rajasthan	Not Accredited	Private university
	NRJJO24	All India Institute of Medical Sciences (AIIMS)	Jodhpur	Rajasthan	Not Accredited	UGC approved Institute of National Importance
	NRJK25	Career Point University	Kota	Rajasthan	Not Accredited	Private university
	NRJC26	OPJS University	Churu	Rajasthan	Not Accredited	Private university
	NRJJA27	Poornima University	Jaipur	Rajasthan	Not Accredited	Private university
	NRJJO28	Jodhpur School of Public Health	Jodhpur	Rajasthan	Not Accredited	Private university
	NRJJO29	Maulana Azad University	Jodhpur	Rajasthan	Not Accredited	Private university
	NRJA30	Sunrise University	Alwar	Rajasthan	Not Accredited	Private university
	NRJJH31	Jagdishprasad Jhabarmal Tibrewala University	Jhunjhunu	Rajasthan	Not Accredited	Private university

	NRJJH32	Birla Institute of Technology and sciences	Jhunjhunu	Rajasthan	Accredited	Deemed university
	NRJJA33	Rajasthan University of Health Sciences	Jaipur	Rajasthan	Not Accredited	State university
	NDLWD34	Rajiv Gandhi Paramedical Institute	-	Delhi	Not Accredited	Not approved
	NDLSD35	Athar Institute of Health and Management Studies	-	Delhi	Not Accredited	Not approved
**NORTH -EAST**	NENLD1	Global Open University	Dimapur	Nagaland	Not Accredited	Private university
	NESKG2	Sri Ramaswamy Memorial University Sikkim (SRMUS)	Gangtok	Sikkim	Not Accredited	Private university
	NEMNI3	Bir Tikendrajit University	Imphal	Manipur	Not Accredited	Private university
	NEMLS4	Martin Luther Christian University	Shillong	Meghalaya	Not Accredited	Private university
	NEMLS5	Indian Institute of Public Health Shillong	Shillong	Meghalaya	Not Accredited	Private university
	NEARP6	Himalayan University	Papum Pare District	Arunachal Pradesh	Not Accredited	Private university
	NEARWS7	North East Frontier Technical University (NEFTU)	West Siang Distt	Arunachal Pradesh	Not Accredited	Private university
**EAST**	EJHR1	YBN University	Ranchi	Jharkhand	Not Accredited	Private university
	EWBK2	Institute of Management Study	Kolkata	West Bengal	Accredited	State university
	EWBK3	NSHM Knowledge campus	Kolkata	West Bengal	Accredited	State university
	EWBK4	KPC Medical College and Hospital	Kolkata	West Bengal	Not Accredited	State university
	EWBK5	Haldia Institute of Management	Kolkata	West Bengal	Accredited	State university
	EODK6	Kalinga Institute of Industrial Technology University (KIIT)	Bhubaneswar	Odisha	Accredited	Deemed university

	EODK7	Asian Institute of Public Health (AIPH)	Bhubaneswar	Odisha	Not Accredited	Private university
	EODK8	Indian Institute of Public Health Orrisa	Bhubaneswar	Odisha	Accredited	State university
	EODK9	ICMR-Regional Medical Research Centre	Bhubaneswar	Odisha	Accredited	State university
	EODC10	Ravenshaw University	Cuttack	Odisha	Accredited	State university
	EODC11	Sri International College	Cuttack	Odisha	Accredited	State university
**CENTRAL**	CCHR1	All India Institute of Medical Sciences (AIIMS)	Raipur	Chhattisgarh	Not Accredited	UGC approved Institute of National Importance
	CUPN2	Amity Institute of Public Health & Hospital Administration	Noida	Uttar Pradesh	Accredited	Private university
	CUPP3	Shalom Institute of Health and Allied Sciences (SIHAS)	Allahabad	Uttar Pradesh	Not Accredited	Deemed university
	CUPG4	Noida School of Allied Health Sciences	Gautam Buddh Nagar district	Uttar Pradesh	Not Accredited	Private university
	CUPL5	Maharishi University of Information Technology	Lucknow	Uttar Pradesh	Not Accredited	Private university
	CUPL6	Institute of Natural Sciences & Humanities	Lucknow	Uttar Pradesh	Accredited	Private university
	CUPA7	Dr Ram Manohar Lohia Avadh University (RMLAU)	Ayodhya	Uttar Pradesh	Not Accredited	State university
	CUPGh8	ICRI- Ghaziabad campus (executive mph)	Ghaziabad	Uttar Pradesh	Not Accredited	Private university
	CUPL9	Dr. Giri Lal Gupta Institute of Public Health and Public Affair (GLGIOPH)	Lucknow	Uttar Pradesh	Accredited	State university
	CUPB10	Bareilly International University	Bareilly	Uttar Pradesh	Not Accredited	Private university

	CUKD11	All India Institute of Medical Sciences (AIIMS)	Rishikesh	Uttarakhand	Not Accredited	UGC approved Institute of National Importance
	CUKP12	Maharaja Agrasen Himalayan Garhwal University (MAHGU)	Pauri Garhwal district	Uttarakhand	Not Accredited	Private university
	CUKD13	Combined PG Institute of Medical Sciences and Research Private	Dehradun	Uttarakhand	Not Accredited	Central university
	CUKH14	Sri Ram Educational and Welfare Trust-Bhartiya Mahavidyalaya	Roorkee	Uttarakhand	Not Accredited	State university
	CUKD15	Uttaranchal College of Health Sciences	Dehradun	Uttarakhand	Accredited	Private university
	CUKD16	ICRI-Bhopal campus	Bhopal	Madhya Pradesh	Not Accredited	Private university
	CMPB17	Rabindranath Tagore University	Raisen district	Madhya Pradesh	Accredited	Private university
	CMPR18	Azim Premji University Bhopal campus	Bhopal	Madhya Pradesh	Not Accredited	Private university
	CMPB19	Swami Vivekanand University	Sagar district	Madhya Pradesh	Not Accredited	Private university
	CMPS20	Sai group of institutions	Dehradun	Uttarakhand	Not Accredited	Not approved
**WEST**	WGJS1	P P Savani University	Surat	Gujarat	Not Accredited	Private university
	WGJV2	Parul University	Vadodara	Gujarat	Accredited	Private university
	WGJAh3	Indian Institute of Public Health Gandhinagar	Ahmedabad	Gujarat	Not Accredited	Private university
	WGJAn4	Bhaikaka University	Anand district	Gujarat	Not Accredited	Private university
	WMHPu5	Symbiosis Institute of Health Sciences	Pune	Maharashtra	Accredited	Deemed university
	WMHW6	Datta Meghe Institute of Higher Education and Research	Wardha	Maharashtra	Accredited	Deemed university
	WMHAh7	Pravara Institute of Medical Sciences University	Ahmednagar	Maharashtra	Not Accredited	Deemed university

	WMHPu8	MIT World Peace University (MIT WPU)	Pune	Maharashtra	Not Accredited	Private university
	WMHPu9	Dr D Y Patil Vidyapeeth	Pune	Maharashtra	Accredited	Deemed university
	WMHS10	Krishna Institute of Medical Sciences (KIMS)	Satara district	Maharashtra	Accredited	Deemed university
	WMHTh11	Padmashree D Y Patil University	Mumbai	Maharashtra	Accredited	Deemed university
	WMHN12	Maharashtra University of Health Sciences	Nashik	Maharashtra	Not Accredited	State university
	WMHRa13	MGM School of Biomedical Sciences	Mumbai	Maharashtra	Accredited	Deemed university
	WMHP14	Savitribai Phule Pune University	Pune	Maharashtra	Not Accredited	State university
	WMHM15	National Institute of Public Health Training and Research ministry of health and family welfare	Mumbai	Maharashtra	Not Accredited	State university
	WMHM16	ICRI-Mumbai	Mumbai	Maharashtra	Not Accredited	Private university
	WMHM17	Indian School of Technology and Management	Mumbai	Maharashtra	Not Accredited	Not Approved
**SOUTH**	SPYP1	Jawaharlal Institute of Postgraduate Medical Education & Research (JIPMER)	-	Puducherry	Not Accredited	UGC approved Institute of National Importance
	SPYP2	Vinayaka Mission's Research Foundation AVMC campus	-	Puducherry	Accredited	Deemed university
	STSRR3	University of Hyderabad	Hyderabad	Telangana	Accredited	Central university
	STSRR4	Indian Institute of Public Health Hyderabad	Hyderabad	Telangana	Not Accredited	State university
	SKLT5	Sree Chitra Tirunal Institute for Medical Sciences and Technology	Thiruvananthapuram	Kerala	Not Accredited	UGC approved Institute of National Importance

	SKLE6	Amrita Institute of Medical Sciences and Research Centre	Kochi	Kerala	Accredited	Deemed university
	SKLKA7	Central University of Kerala	Kasaragod district	Kerala	Accredited	Central university
	SKLKO8	CENTRE for Professional and Advanced Studies	Kottayam	Kerala	Not Accredited	State university
	SKLT9	Global Institute of Public Health	Thiruvana nthapuram	Kerala	Not Accredited	State university
	STNV10	Christian Medical College	Vallore	Tamil Nadu	Not Accredited	UGC approved Institute of National Importance
	STNC11	Sri Ramachandra Institute of Higher Education and Research (SRIHER)	Chennai	Tamil Nadu	Accredited	Deemed university
	STNCh12	SRM Institute of Science and Technology (SRMIST)	Chennai	Tamil Nadu	Accredited	Deemed university
	STNS13	Vinayaka Mission's Research Foundation Salem campus	Salem	Tamil Nadu	Accredited	Deemed university
	SKAM14	JSS Academy of Higher Education and Research	Mysore	Karnataka	Accredited	Deemed university
	SKAB15	Jawaharlal Nehru Medical College	Belagavi	Karnataka	Accredited	Deemed university
	SKABu16	Dayanand Sagar University	Bangalore	Karnataka	Not Accredited	Private university
	SKADk17	Edward & Cynthia Institute of Public Health	Mangaluru	Karnataka	Accredited	Deemed university
	SKAB18	MS Ramaiah University of Applied Sciences Gnanagangothri campus	Bangalore	Karnataka	Not Accredited	Private university
	KABu19	Padmashree institute of public health	Bangalore	Karnataka	Not Accredited	State university
	SKAB20	National Institute of Mental Health and Neurosciences (NIMHANS)	Bangalore	Karnataka	Not Accredited	UGC approved Institute of National Importance

	SKAK21	Sri Devraj Urs Academy of Higher Education and Research	Kolar	Karnataka	Accredited	Deemed university
	SKAG22	Karnataka State Rural Development and Panchayat Raj University (KSRDPRU)	Gadag district	Karnataka	Not Accredited	State university
	SKAU23	Prasanna School of Public Health	Udupi	Karnataka	Accredited	Deemed university
	SKADk24	KS Hegde Medical Academy	Mangaluru	Karnataka	Accredited	Deemed university
	SAPC25	The Apollo University	Chitoor	Andhra Pradesh	Not Accredited	Private university
	SAPC26	Om Sri Gayatryviswakarma University	Chittoor	Andhra Pradesh	Not Accredited	Not Approved
